# The Effects of Silica Fume and Superplasticizer Type on the Properties and Microstructure of Reactive Powder Concrete

**DOI:** 10.3390/ma16206670

**Published:** 2023-10-13

**Authors:** František Šoukal, Luboš Bocian, Radoslav Novotný, Lucie Dlabajová, Nikola Šuleková, Jan Hajzler, Ondřej Koutný, Martina Drdlová

**Affiliations:** 1 Faculty of Chemistry, Brno University of Technology, Purkyňova 118, 612 00 Brno, Czech Republic; 2 Bogges s.r.o., Hněvkovského 30/65, 617 00 Brno, Czech Republic; 3 Research Institute of Building Materials, Hněvkovského 30/65, 617 00 Brno, Czech Republic

**Keywords:** reactive powder concrete, ultra-high-performance concrete, silica fume, superplasticizer, microstructure, pozzolanic reaction

## Abstract

This paper deals with the optimization of reactive powder concrete mixtures with respect to the addition of silica fume and the type of polycarboxylate superplasticizer used. First, the properties of reactive powder concrete with eight different commercial polycarboxylate superplasticizers were tested in terms of workability, specific weight, and mechanical properties. It was found that different commercially available superplasticizers had significant effects on the slump flow, specific weight, and compressive and flexural strengths. The optimal superplasticizer (BASF ACE430) was selected for further experiments in order to evaluate the influences of silica fume and superplasticizer content on the same material properties. The results showed that the silica fume and superplasticizer content had considerable effects on the mini-cone slump flow value, specific weight, flexural and compressive strengths, and microstructure. There were clearly visible trends and local minima and maxima of the measured properties. The optimal reactive powder concrete mixture had a composition of 3.5–4.0% superplasticizer and 15–25% silica fume.

## 1. Introduction

The first mention of the term reactive powder concrete (RPC) in the literature is believed to have come from the work of its inventors, P. Richard and M. H. Cheyrezy. RPC can be considered as one of the latest types of ultra-high-performance concrete (UHPC), and perhaps the one that can be considered the most important to this day. It can be found in the literature that the term UHPC is sometimes used solely for RPC, but this is not correct, because RPC is only one type of UHPC. The extraordinary properties of RPCs, such as very high compressive strength (up to 800 MPa) or the relatively high homogeneity of the matrix, lie mainly in the optimization of the particle size distribution of all the solid components, the elimination of coarse aggregates larger than 600 µm, the use of very low water-to-cement ratios ranging from 0.18 to 0.30, very low porosity, and the use of relatively high amounts of superplasticizer. The ductility, fracture energy, flexural strength, and compressive strength were previously enhanced by the incorporation of short steel fibers with varying sizes and geometry. The use of synthetic fibers in RPC was also proven to be beneficial, albeit limited compared to the use of steel fibers. The term reactive powder refers to the possibility of chemical reactions between the components of the mixture. Quite remarkable projects such as Mauves sur Loire Bridge and the Central Bank of Iraq were realized with the use of RPC [1,2,3,4,5,6,7,8,9,10,11,12,13].

Concrete is one of the most widely used materials in the world. However, the main disadvantage of concrete is that its production contributes a fair amount to the production of CO_2_. This leads to the utilization of industrial byproducts, which would otherwise be considered as waste, in concrete to lower its cost or enhance its properties. One of such supplementary cementitious materials (SCMs) is silica fume, which is mostly a byproduct of the production of silicon and ferrosilicon. It is available in powder form as spherical particles that are generally around 200 nm in diameter. The addition of silica fume into the mixture is also beneficial to the properties of hardened concrete, in addition to making it more environmentally friendly because of the lower content of cement in the mixture. The enhancement of the properties of hardened concrete has been attributed to the decrease in Ca(OH)_2_ and the increase in the C-S-H gel with the addition of silica fume, greatly increasing the density of the microstructure of the matrix and decreasing its porosity. Consequently, the decrease in Ca(OH)_2_ was reported in the literature [14,15,16,17,18,19,20,21,22] to improve the interfacial zone if steel fibers are incorporated into the matrix, which is considered to be a synergistic effect between the steel fibers and the silica fume.

As has clearly been demonstrated many times before, the mechanical properties of RPC significantly increase with the addition of silica fume. It is also one of the most abundant SCMs today, and it was proven to significantly decrease the Ca/Si ratio and possibly decrease Ca(OH)_2_ to the point that it cannot be found in the microstructure anymore. The addition of silica fume can also increase the packing density of RPC, and the interfacial zone between the aggregate and the paste is also positively influenced by the addition of silica fume into the mixture. The addition of silica fume to RPC has even been observed to positively affect the RPC’s behavior under high-temperature conditions. All of these advantages were attributed to the pozzolanic reaction of silica fume and to the acceleration of cement hydration with the addition of silica fume. The low workability with the increased addition of silica fume can be considered to be the main disadvantage of RPC mixtures with silica fume compared to SCM mixtures containing fly ash or ground granulated blast furnace slag [23,24,25,26,27,28].

Superplasticizers, also known as high-range water reducers, are admixtures for cementitious materials that produce mixtures with low w/c ratios and high workability. These admixtures have also been shown to be beneficial for decreasing the porosity of the final product (by means of decreasing the w/c ratio). Superplasticizers are water-soluble polymers with anionic groups that can have both relatively low and relatively high molecular weights. However, the superplasticizers available to customers are often formulations consisting of two or more types of polymers. It should be noted that the lower the molecular weight of such a polymer, the more air is entrapped during mixing. Superplasticizers have also been proven to disperse cement particles, that would otherwise be agglomerated, by decreasing the attractive forces between them and decreasing the force that causes low flow in the mixture. Steric hindrance has also become a major element of the plasticization mechanism with the advent of comb-shaped copolymers such as polycarboxylates (PCs). Polycarboxylates can be molecules with quite complex structures, which manifest themselves in the effects of the superplasticizer. For example, PCs with the carboxylic group exhibit a high rate of initial adsorption on cement particles but induce poor slump retention. On the other hand, sulfonic and phosphonic groups can exhibit rather different behaviors, but this also depends on a number of different factors. More branched PCs work better for cement dispersion and have lower retardation effects on cement hydration. This is similar to the fact that the longer the PC backbone, the lower the retardation effect and the higher the flowability. This is also valid for the molecular weight of the PC backbone in terms of flowability. However, it is beyond the scope of this study to describe the dependence of the effects of the PC superplasticizer on its structure in detail. The ideal dosage of the superplasticizer depends mainly on the surface quality of the cement and the surface of the particles, so relatively high dosages of superplasticizer are needed for mixtures with very fine materials [29,30,31,32,33].

There are relatively limited studies in the literature on the optimization of the polycarboxylate superplasticizer type and content in RPC. It is also worth mentioning that not only the type and manufacturer of superplasticizer, but also the type of cement and the water-to-cement (w/c) or water-to-binder (w/b) ratio have been proven to affect each other, so all of these facts should be properly described in the literature. The superplasticizers and their contents also have significant impacts on properties such as particle packing [34,35,36,37,38].

The main objectives of this paper are the selection of the most suitable superplasticizer for a designed RPC mixture and the determination of the optimal content of superplasticizer with connection to the silica fume content to obtain the best workability of fresh concrete and the best mechanical properties and density. The effects of different additions of silica fume with different additions of PC superplasticizer on the properties and microstructure of RPC has not been thoroughly studied anywhere yet. Furthermore, the topic of the influence of PC superplasticizers in the context of a dry mixture has not been covered in the literature. The measured values of the investigated properties were plotted against the superplasticizer and silica fume contents in the matrix. Based on the above-mentioned literature review, the research hypothesis assumes that the optimal superplasticizer and its content connected with optimal silica fume content can be determined within their measured ranges. Furthermore, each mixture design requires a specific type of superplasticizer to obtain the desired material properties. The RPC mixture was designed for utilization in panels for the ballistic protection of critical infrastructure objects; therefore, the maximal compressive and flexural strengths and the specific weight at a suitable workability are required. The ballistic protection performance will be developed and tested within the following works.

There are also two aspects that must be considered. The overdosage of a superplasticizer could significantly retard the hydration of Portland cement, and a higher content of silica fume could decrease the hydration heat; therefore, the hydration route must be examined via isothermal calorimetry.

## 2. Materials and Methods

### 2.1. Preparation of Specimens and Slump Flow Test

All samples were prepared in the same way, regardless of the type of mixture. The mixture compositions for the optimization of the type of superplasticizer are listed in Table 1. Please note that the BASF Melflux 2651F superplasticizer was supplied and added to the mixture as a dry powder. Each superplasticizer was dosed so that the dry content of each superplasticizer was exactly 7.2 g and the water content was the same for all mixtures. The mixture compositions for the optimization of the amounts of silica fume and superplasticizer are listed in Table 2. Please note that silica fume was added to the mixtures as a substitute for cement. The water-to-binder ratio was kept constant. The MasterGlenium ACE430 superplasticizer was chosen for the mixes to optimize the contents of silica fume and superplasticizer due to the reasons stated in the results and discussion. The mixtures for the optimization of the silica fume and superplasticizer contents are designated as SFx-Spy, where x represents the percentage of silica fume and y represents the percentage of dry content of the superplasticizer.

The mixing was performed as follows. First, cement, silica fume, and fine sand were weighted into a mixer bowl. The superplasticizer was then added to 250 mL of water. The amount of water shown with the plus sign in the tables was added to the mixture with 20 mL syringes. After that, the dry mixture was stirred thoroughly in a mixer (Artisan series 5, KitchenAid, Benton Harbor, MI, USA) at the lowest possible stirring speed for 1 min to obtain a homogeneous mixture. After that, water with superplasticizer was slowly added to the mixture. Then, the mixture was slowly stirred until plasticization; next, the stirring speed was adjusted to the medium stirring speed. The remaining water was added to the mixture after 5 min of mixing. The mixing continued for another 5 min. After that, the mini-cone slump flow test was performed.

The mini-cone slump flow test was performed with mini cone with a height of 6 cm, a smaller opening diameter of 7 cm, and a larger opening diameter of 10 cm. The mini cone was filled with the mixture and then lifted up. After 30 s from having lifted the cone, two values for slump flow were measured using measuring tape. Those two values were measured roughly perpendicular to each other. The slump flow itself was then the average of those two values. The specimens for the testing of mechanical properties were then prepared by pouring the mixture into the molds. Six samples with dimensions of 4 × 4 × 16 cm^3^ were prepared for determination of mechanical properties at 7 and 28 days.

### 2.2. Evaluation of Mechanical Properties

The testing of mechanical properties of specimens with dimensions of 4 × 4 × 16 cm^3^ at 7 d and 28 d was carried out according to the ČSN EN ISO 196-1 standard [39]. The flexural strength was evaluated using Instron 5895 with a 250 kN load cell. The span length was 100 mm. The preload was 5 kN, and the crosshead speed for the preload was 3 mm/min, after which the crosshead speed was changed to the loading rate of 0.08 kN/s. A compressive strength test was performed on each end of the specimen after the flexural strength test. This test was carried out using a concrete testing machine with a 3000 kN load cell, DESTTEST 3310 (BetonSystem, Brno, CZ, Czech Republic). The loaded area was always 1600 mm^2^, and the loading rate was 2.4 kN/s. The mechanical properties were measured for six specimens at the ages of 7 and 28 days.

### 2.3. Isothermal Calorimetry

The measurement of isothermal calorimetry was performed using the TAM AIR isothermal calorimeter (TA Instruments, New Castle, DE, USA). The testing was conducted with selected RPC mixtures with the same compositions as in Table 1 and Table 2. For each measurement, 50 g of the mixture was made, but only 10 ± 0.001 g of the mixture was placed in the 20 mL plastic ampoule. The reference material was demineralized water in the amount that corresponded to the thermal capacity of the RPC mixture. Two measurement series, one for different dosages of superplasticizer and one for different dosages of silica fume, were performed.

### 2.4. Evaluation of Specific Weight

The evaluation of the specific weight was performed after 28 d. The samples were weighted prior to the test of mechanical properties. The specific weight was obtained by dividing the weight of the sample by the volume of the sample.

### 2.5. Scanning Electron Microscopy

The thin cross sections of the RPC specimens were first cut with a diamond saw from the specimens used for the testing of the mechanical properties. These specimens were then flooded with epoxy resin and polished until a highly polished surface was obtained. Then, they were analyzed with JEOL JSM-7600F at 15 kV. The Ultim Max 100 (Oxford Instruments, Abingdon-on-Thames, UK) EDS spectrometer was used for the EDS analysis of RPC mixtures with the addition of silica fume. The elemental maps showing the distribution of the significant chemical elements (Al, Ca, and Si) based on the EDS measurement were prepared.

## 3. Results and Discussion

### 3.1. Mechanical Properties, Specific Weight, and Slump Flow

#### 3.1.1. Different Types of Superplasticizers

**Figure 1:**
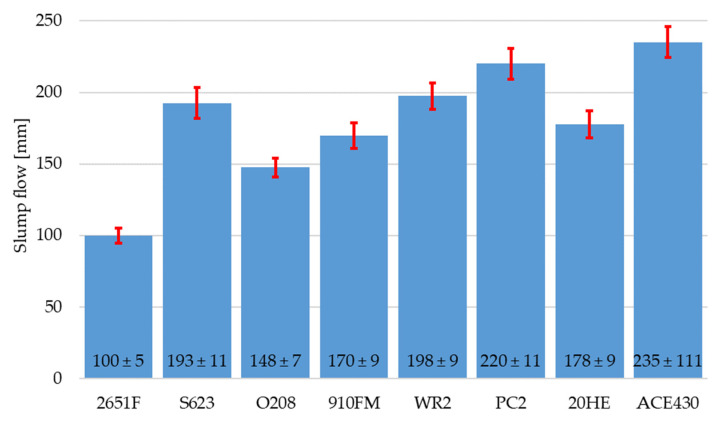
Slump flow of RPC mixtures with different polycarboxylate superplasticizers.

**Figure 2:**
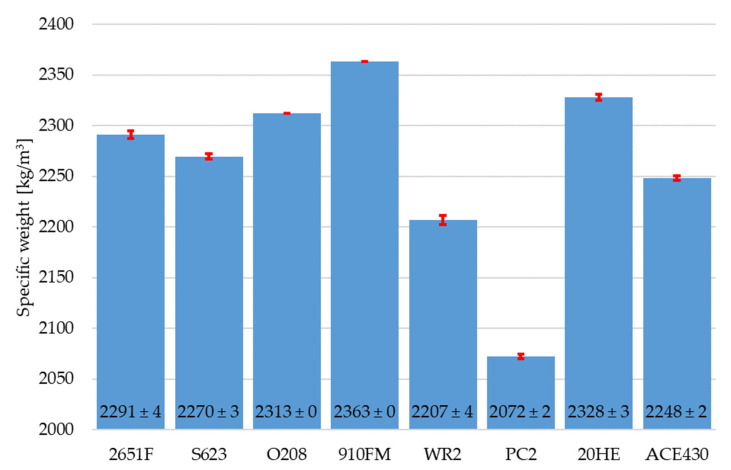
Specific weight of RPC mixtures with different polycarboxylate superplasticizers.

The slump flow of the RPC mixtures with different polycarboxylate superplasticizers is shown in Figure 1. The standard deviation for the slump flow was calculated from four measured values for each mixture. The specific weight values of the RPC mixtures with different polycarboxylate superplasticizers are presented in Figure 2. The standard deviation for the specific weight was calculated from six values for each mixture. It can be clearly seen that the ACE430 superplasticizer provided the fresh mixture with the highest slump flow value of 235 ± 11 mm. A similar value was also achieved with the PC2 superplasticizer with a slump flow of 220 ± 11 mm, but the use of the PC2 superplasticizer led to the lowest specific weight of 2072 ± 2 kg/m^3^. This was probably caused by the air entrainment in the fresh mixture. On the other hand, the use of ACE430 did not lead to the highest specific weight, but it provided a significantly higher workability, which is why this superplasticizer was chosen for further experiments. The highest value of specific weight (2360 ± 0 kg/m^3^) was provided by the 910FM superplasticizer, but it also provided significantly lower workability compared to the workability achieved with the ACE430 superplasticizer, which could negatively affect the properties of this mixture if fibers or some other type of reactive or filler material were added to the RPC mixture.

**Figure 3:**
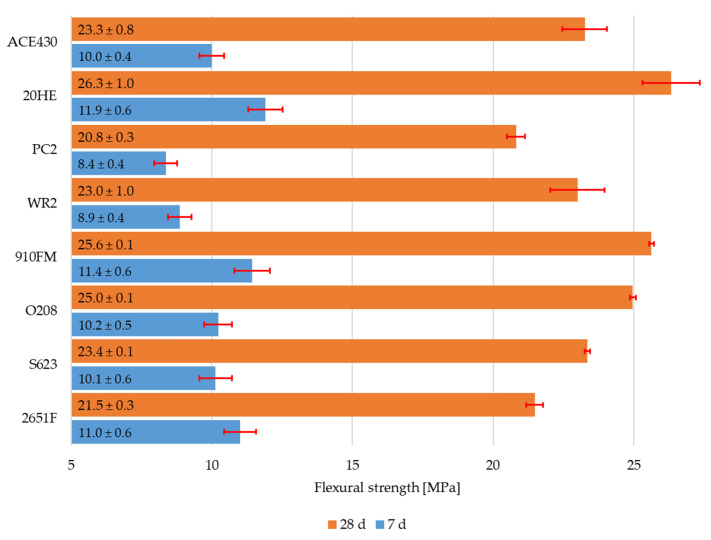
Flexural strength of RPC mixtures with different polycarboxylate superplasticizers after 7 d and 28 d.

**Figure 4:**
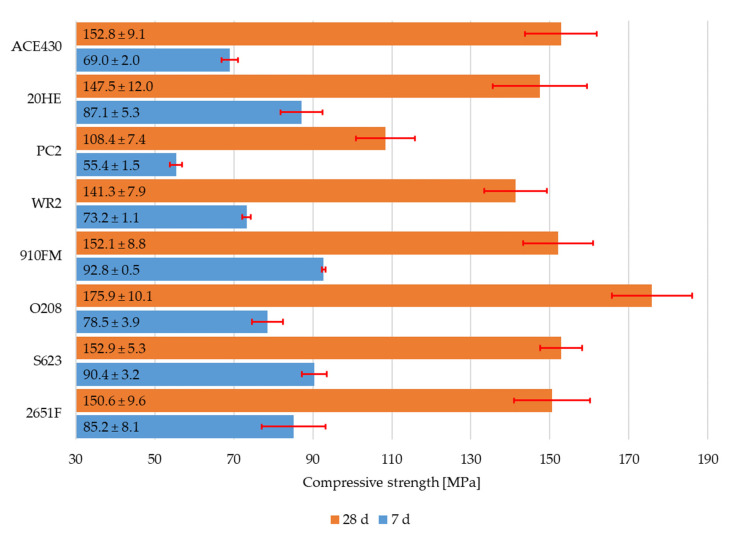
Compressive strength of RPC mixtures with different polycarboxylate superplasticizers after 7 d and 28 d.

The flexural strength and the compressive strength of RPC mixtures with different types of polycarboxylate superplasticizers can be seen in Figure 3 and Figure 4. The standard deviation was calculated from six values for flexural strength and from twelve values for compressive strength. The highest value of flexural strength after 7 d and 28 d was provided by the 20HE superplasticizer. The values were 11.9 ± 0.6 MPa and 26.3 ± 1.0 MPa, respectively. However, its workability was lower than that of the mixture with the ACE430 superplasticizer even though the specific weight was slightly higher. This was also true for the mixtures with the 910FM, O208, and S623 superplasticizers, which all had slightly higher flexural strengths after 28 d in comparison to the mixture with ACE430, but they did not exhibit such high workability, which is not desirable, especially if fibers are added into the mixture.

On the other hand, almost all compressive strengths after 28 d reached roughly the same value, except for the highest compressive strength after 28 d (175.9 ± 10.1 MPa), which was achieved by the RPC mixture with the O208 superplasticizer. This could be attributed to the higher specific weight of this RPC mixture. The RPC mixture with the PC2 superplasticizer showed the worst results in both the flexural and compressive strengths after 7 and 28 d. This could be attributed to the air entrained in the mixture, which contributed to the presence of defects, resulting in increased porosity. The effect of air entrainment could be observed mainly in the samples after compressive strength testing, even though the flexural and compressive strengths were not affected. These assumptions about the mechanical performance of the PC2 superplasticizer are supported by the literature [36]. The ACE430 superplasticizer was evaluated as the optimal superplasticizer for future studies due to the overall good results in terms of all of the properties mentioned above.

#### 3.1.2. Different Dosages of Silica Fume and Superplasticizer

**Figure 5:**
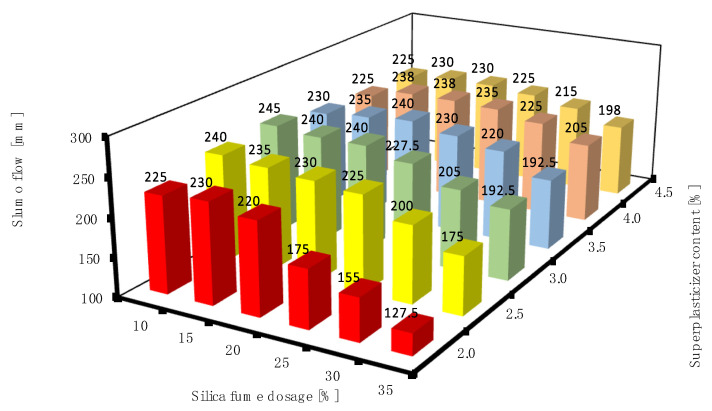
Slump flow of RPC mixtures with different dosages of silica fume and superplasticizer.

Figure 5 shows the slump flow of all produced RPC mixtures. Please note that the standard deviations were not introduced into all 3D column graphs to keep the clarity. It can be clearly seen that the more silica fume in the mixture, the lower the value of the slump flow. This could be attributed to the high specific surface of silica fume particles. However, the addition of a superplasticizer could efficiently act against this. The highest values of the slump flow were achieved for silica fume dosages between 10% and 20% and for superplasticizer contents of 3 to 4%. However, satisfactory values of the slump flow seemed to be achieved with almost all silica fume dosages and superplasticizer contents in addition to mixtures with silica fume ranging from 30 to 35% and superplasticizer contents ranging between 2 and 4.5%, which yielded the lowest slump flow values. The value of the slump flow of the SF25/SP2.0 mixture was also relatively low compared to the other results. The facts that the diameter of the spread of the mini-cone slump increased with the increase in the superplasticizer content, and the workability decreased due to the incorporation of silica fume, are both in agreement with the previous literature [6,38].

**Figure 6:**
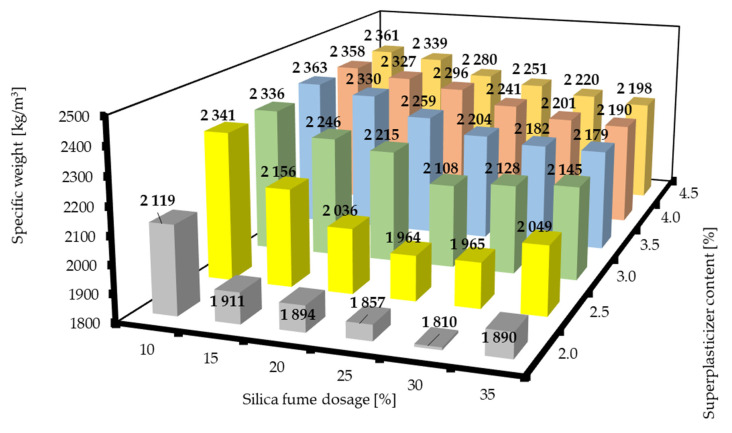
Specific weights of RPC mixtures with different dosages of silica fume and superplasticizer.

The specific weights of RPC mixtures with different dosages of silica fume and superplasticizer are presented in Figure 6. A clear trend in terms of the superplasticizer content can be observed. The value of the specific weight increased with the content of the superplasticizer. There was, however, a rather different relation between the specific weight and the addition of silica fume. The lowest specific weight values were achieved for the silica fume dosages of 25–30% regardless of the superplasticizer content. Then, an upward trend was observed with the silica fume dosage increasing or decreasing from the values of 25 to 30% regardless of the superplasticizer content. Apparently, the more superplasticizer used, the higher the value of the specific weight, but this effect was less pronounced with higher contents of superplasticizer. This effect could be attributed to possible particle packing problems if the superplasticizer does not properly distribute the cement grains at lower superplasticizer contents. However, it seems that if silica fume has a beneficial effect on mechanical properties, it is mainly caused by the enhancement of the microstructure rather than the increase in the specific weight. Apparently, the compromise between good workability and the highest possible specific weight lies in the region of the mixture composition of SF20/SP3.5.

**Figure 7:**
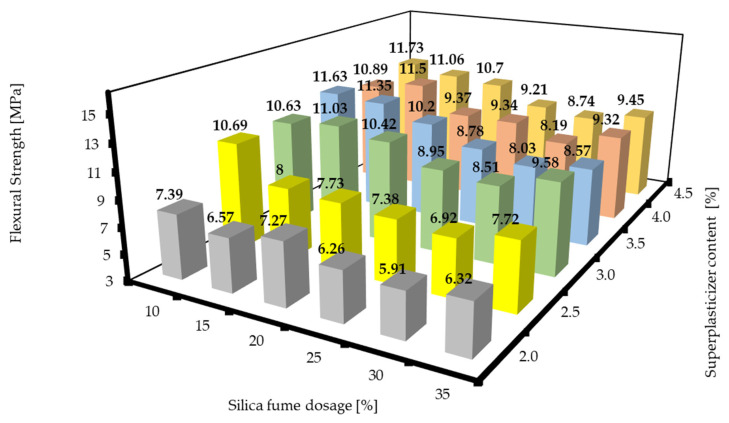
Flexural strengths of RPC mixtures with different silica fume dosages and superplasticizer contents after 7 d.

**Figure 8:**
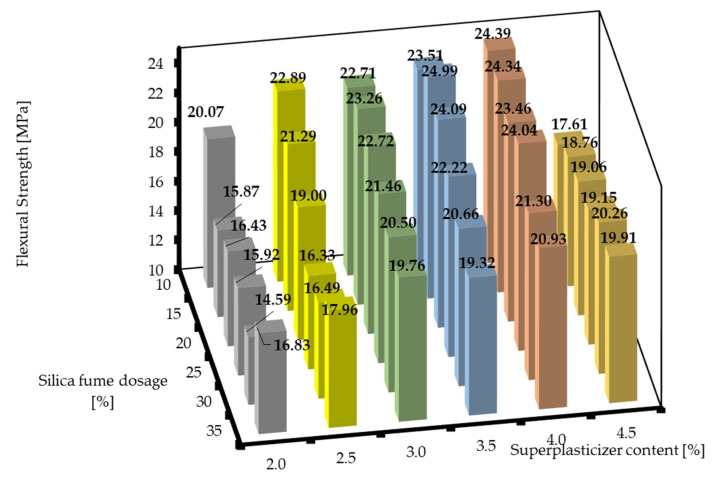
Flexural strengths of RPC mixtures with different silica fume dosages and superplasticizer contents after 28 d.

The flexural strengths of the RPC mixtures with different dosages of silica fume and superplasticizer contents after 7 d and 28 d can be seen in Figure 7 and Figure 8. It is clearly visible that higher contents of superplasticizer and lower contents of silica fume tended to reach the highest values. This could mean that there was no significant acceleration of hydration using silica fume in earlier stages and that there could be some deceleration due to the amount of superplasticizer used (see Section 3.2).

The situation was totally different for the flexural strength after 28 d. It is clearly shown in Figure 8 that the highest flexural strengths were obtained when the RPC mixtures had the composition around SF10/SP3.5 to SF20/SP4.0. The mixtures with lower dosages of silica fume and superplasticizer showed significantly lower flexural strengths after 28 d. However, there was a steep decline in the flexural strength at 4.5% of superplasticizer, which means that the superplasticizer dosages greater than 4.0% significantly decreased the rate of pozzolanic reaction. However, an optimal plateau for the silica fume dosage was also presented. This could be attributed to the fact that there should be a higher ratio of reacted silica fume in the RPC mixture with lower amounts of silica fume, thus contributing to the mechanical properties of the mixtures by generating more C-S-H gel, rather than acting only as a filler, when added at higher amounts. Other sources offer similar results for the comparison [7,40].

**Figure 9:**
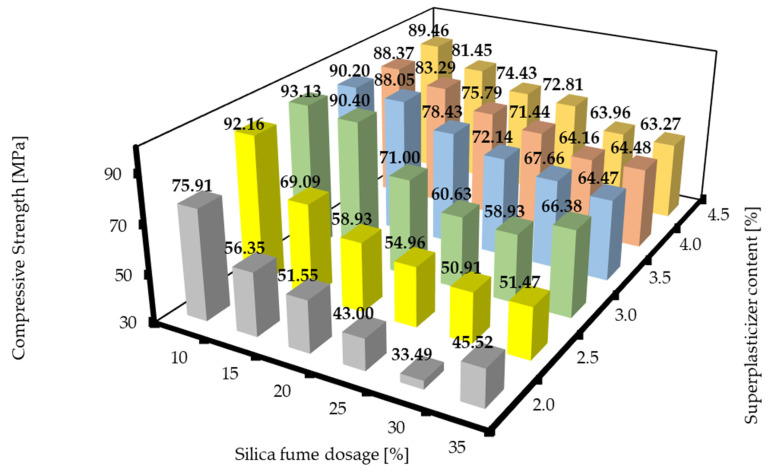
Compressive strengths of RPC mixtures with different silica fume dosages and superplasticizer contents after 7 d.

**Figure 10:**
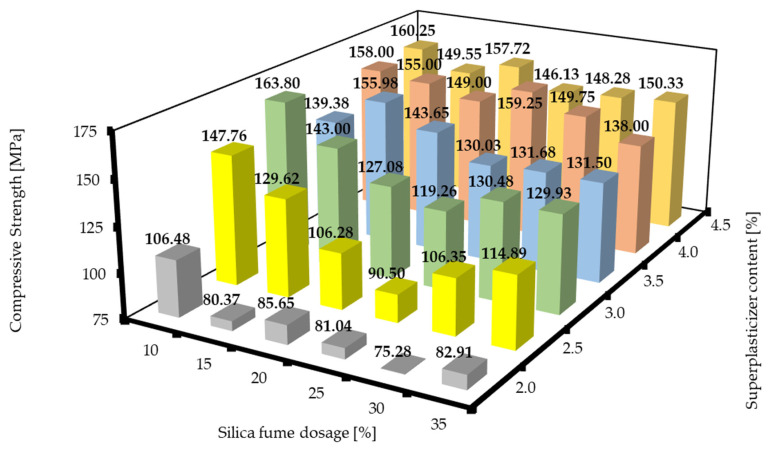
Compressive strengths of RPC mixtures with different silica fume dosages and superplasticizer contents after 28 d.

The compressive strengths of RPC mixtures with different silica fume dosages and superplasticizer contents after 7 d are shown in Figure 9. The compressive strengths of RPC mixtures with different silica fume dosages and superplasticizer after 28 d are shown in Figure 10. Again, the trends were similar to the graphs presented above. There was always a minimum that was observed at the silica fume dosages of about 25 to 30%, and then the values of compressive strength increased with both the decrease and the increase in the silica fume dosage. The values of the compressive strength were always higher with the increase in the superplasticizer content. This could be attributed to the same effects mentioned above for the specific weight. Next, the region that corresponds to the plateau of the flexural strength mentioned above was observed. However, this region was very slightly shifted towards lower superplasticizer dosages, which could possibly be attributed to mild retardation of the hydration process at higher superplasticizer contents. The compressive strengths after 28 d exhibited similar trends, but the plateaus that could be found in Figure 8 and Figure 9 are not visible here, and the highest value of compressive strength after 28 d was achieved by the designed RPC mixture of SF10/SP3.0. However, this RPC mixture did not give the best results in terms of the other characteristic features (properties), so the best mixtures in the overall properties would still be the mixtures in the region around SF10/SP3.5 to SF20/SP4.0. The local minima of the SF10/SP3.5 mixture can be regarded as just a fluctuation of values. These findings correspond with those of other sources [7,40].

### 3.2. Calorimetric Studies

**Figure 11:**
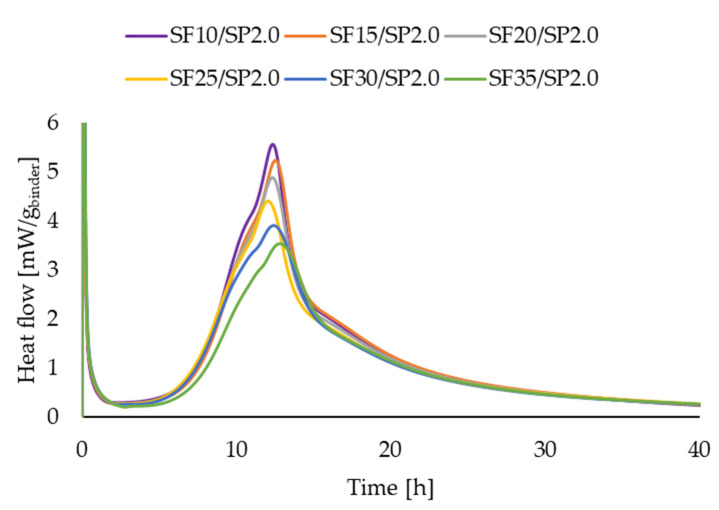
Heat flow measurements for RPC mixtures with increasing superplasticizer additions.

**Figure 12:**
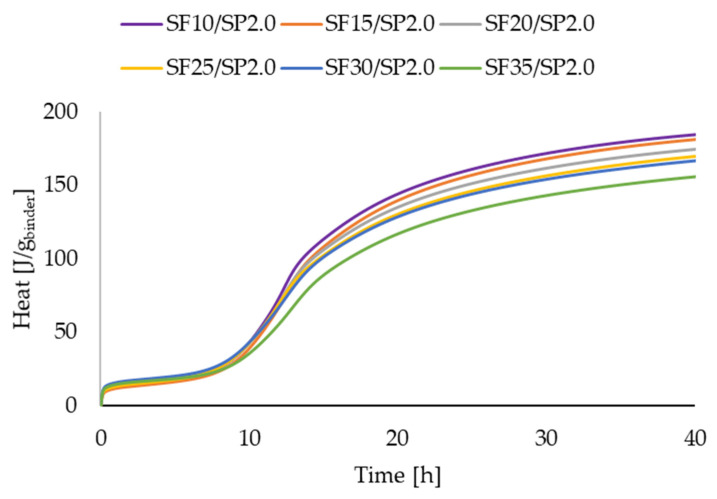
Heat measurements for RPC mixtures with increasing superplasticizer additions.

**Figure 13:**
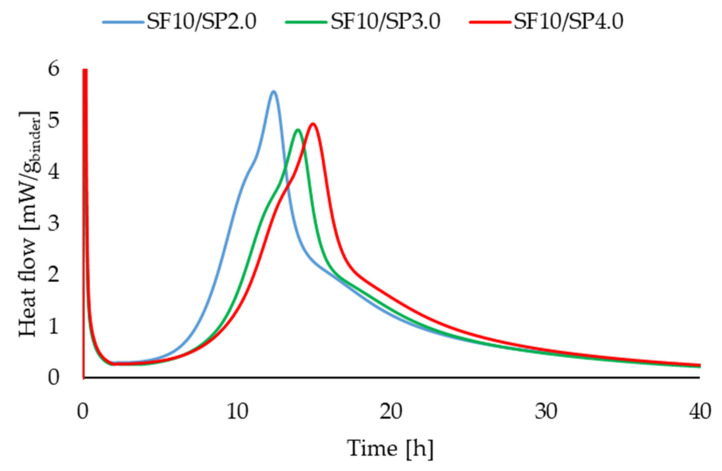
Heat flow measurements for RPC mixtures with constant silica fume addition.

**Figure 14:**
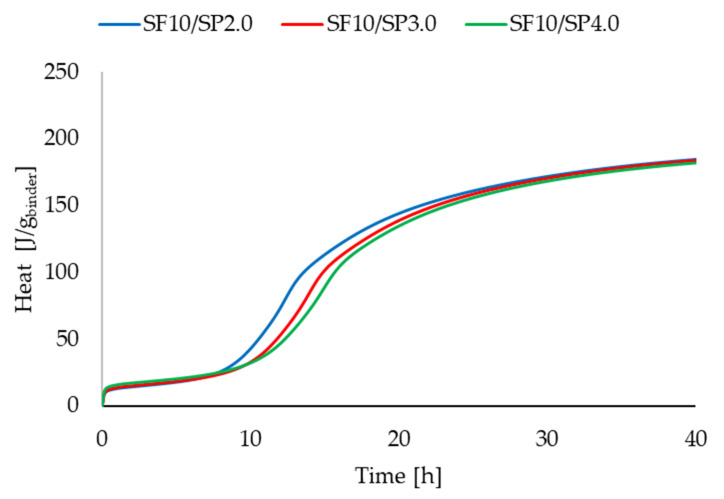
Heat measurements for RPC mixtures with constant silica fume addition.

Figure 11, Figure 12, Figure 13 and Figure 14 present two series of RPC mixtures measured with isothermal calorimetry. In Figure 11 and Figure 12, we can see the calorimetric measurement of the series with a constant 2.0% superplasticizer content and increasing dosage of silica fume. In Figure 13 and Figure 14, the series of the RPC mixtures can be seen with constant silica fume dosages and increasing superplasticizer contents. Please note that the graphs for the superplasticizer content were obtained by measuring the RPC mixtures with an increase of 1.0% in the superplasticizer. Please note that the results in Figure 11, Figure 12, Figure 13 and Figure 14 were calculated; hence, the values are divided by the whole mass of the binder (silica fume + cement).

The measurement of the heat flow and the total heat obtained for the series with a constant superplasticizer dosage can be seen in Figure 11 and Figure 12. It is clearly visible that the addition of silica fume to the RPC mixture reduced both the heat flow and the total heat if the percentage of binder in the mixture was kept constant. This means that the silica fume hydration reaction with Ca(OH)_2_ involved less heat than the cement hydration itself. However, the literature reveals quite an opposite effect of SF addition on the position of the heat flow maximum [41,42,43]. This could be attributed to a better dispersion of SF in the RPC mixture because it has been shown that SF could have a different effect on some properties of cement composites, such as the compressive strength, depending on its dispersion, which is shown in [43].

It can be clearly observed from Figure 13 and Figure 14 that the more superplasticizer added into the RPC mixture, the lesser the heat flow, and less total heat is generated. What was different, however, was the time at which the heat flow reached the maximum. This was probably due to the retardation of hydration with the addition of a superplasticizer. It is not very prominent though, and the retardation was only slight and within two hours from the RPC mixture with 2.0% and 4.0% of the superplasticizer. Comparable results can be found in other articles [44,45].

### 3.3. SEM Observation

**Figure 15:**
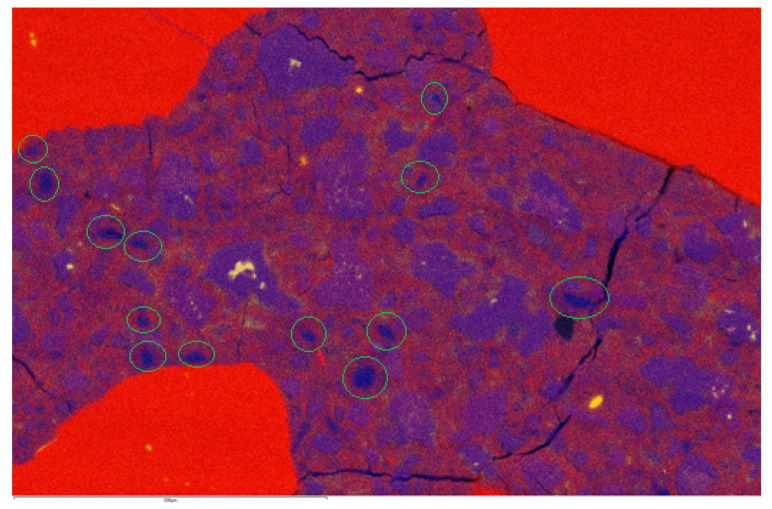
EDS maps of microstructure of RPC mixture with 20% of silica. Yellow—Al; blue—Ca; red—Si. Scale is 100 microns; green circles indicate portlandite.

**Figure 16:**
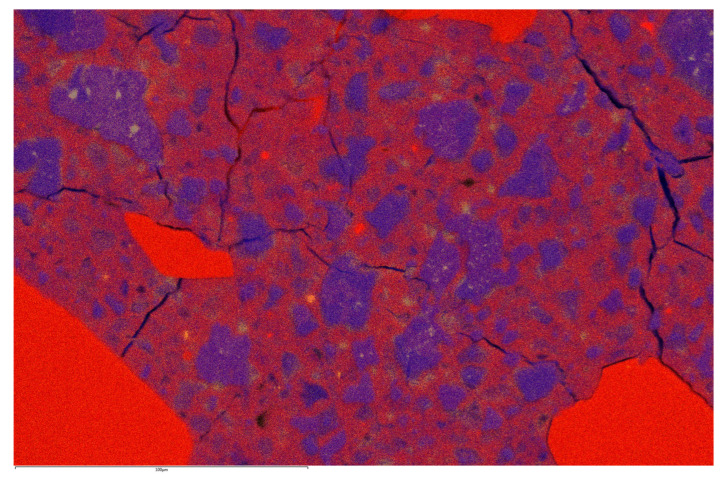
EDS maps of microstructure of RPC mixture with 35% of silica. Yellow—Al; blue—Ca; red—Si; scale is 100 microns.

Figure 15 and Figure 16 present the EDS maps of the microstructures of the RPC mixtures with 20% silica fume and 35% silica fume, respectively. Please, note that the cracks (black lines) are not part of the original microstructure, and they were caused by the vacuum in the SEM. The most important finding was that in Figure 15, there were many small spots that were clearly visible between the cement grains where calcium is located, which means that portlandite was present in these points. Most of the deep blue pockets of portlandite are marked by green circles in Figure 15. However, in Figure 16, it was observed that these deep blue spots of portlandite were no longer located between cement grains. This difference stemmed from the fact that silica fume reacted with excessive calcium ions present in the solution, which produced C-S-H gel instead of crystals of portlandite. It was also proven that the silica fume used in this study could react in this way and thus produce more C-S-H gel, which could be attributed to the beneficial improvement in the properties of the RPC mixtures. This, however, does not say anything about why higher dosages of silica fume were not as beneficial to the properties of RPC mixtures as lower ones, and this fact may lie in the type of C-S-H gel generated at higher doses of silica fume. The description of the same phenomenon has been observed many times before [5,6,26,45].

## 4. Conclusions

The main objectives of the paper were the selection of the most suitable superplasticizer for a designed RPC mixture and the investigation of the optimal contents of superplasticizer and silica fume to obtain the best workability of fresh concrete and the best mechanical properties and high density of hardened RPC. The RPC mixture was designed for utilization in panels for the ballistic protection of critical infrastructure objects; therefore, maximal compressive and flexural strengths and a specific weight at a suitable workability were required.

The authors of the study concluded that various compositions of commercially available PCE superplasticizers provide significantly different workabilities, specific weights, and mechanical properties of RPC. With respect to the above-mentioned planned utilization of RPC, the best compromise of RPC properties (very good mechanical properties and average specific weight at superb workability) was achieved with SP ACE430 (BASF). The best workability from the tested SP portfolio gives an opportunity to even decrease the w/c ratio or optimize/change the solid components in further material development. To sum up this part of the study, it is very crucial to pay close attention to the type of superplasticizer used in the RPC mixture. Attention should also be paid to the fact that one superplasticizer may beneficially affect one or two properties above another, so one must know which property of the resultant RPC mixture is highlighted for the application.

The second objective was to determine the optimal dosages of superplasticizer and silica fume. The optimal dosages determined for RPC in this study were 3.5–4.0% of superplasticizer and 15–25% of silica fume in terms of the mechanical properties, workability, and specific weight. Lower dosages of superplasticizer should be avoided because all the properties mentioned above were significantly reduced at superplasticizer values below 3.0%. It should also be noted that a higher dosage of silica fume does not always result in improved properties, so generally, the optimal dosage must always be determined.

Thirdly, both the superplasticizer content and the silica fume dosage affect the evolution of the hydration heat, as we supposed. Silica fume reduced both the heat flow and the total heat according to the nature of its hydration mechanism. The superplasticizer decreased mainly the heat flow, but it also had some retardation effect in terms of the heat flow maximum. However, this retardation effect was not very pronounced, so there are no significant differences between the superplasticizer dosages overall.

The last conclusion is again consistent with our assumption. The main effect of silica fume on the properties of RPC mixtures lies in its reaction with Ca(OH)_2_ to create the C-S-H gel. This provides a microstructure with significantly less portlandite crystals that was verified using the SEM microstructure analysis.

This paper summarizes the first steps of development of ductile fiber-reinforced RPC for the production of composite panels and modular walls for the temporary and movable ballistic protection of critical infrastructure objects. The conclusion of this paper enables the further development of the RPC matrix and the involvement of fiber reinforcement.

## Figures and Tables

**Table 1:** Composition of all mixtures for optimization regarding the type of PC superplasticizer.

Mixture Designation	Fine Sand According to ČSN EN 196-1 [39] (Filtrační Písky Chlum, CZ)	CEM I 52.5 R-SR 5 White (Aalborg Portland, DE)	Silica Fume RW Füller-Q (Elkem, D)	Superplasticizer	Demineralized Water
ACE430	900 g	675 g	225 g	MasterGlenium ACE 430 (BASF, D)	(114 + 37) mL
				36 mL	
2651F	900 g	675 g	225 g	Melflux 2651F (BASF, D)	(122 + 40) mL
				7.2 g	
S623	900 g	675 g	225 g	Sky 623 (BASF, D)	(100 + 33) mL
				36.0 mL	
O208	900 g	675 g	225 g	Optima 208 (Chryso, FRA)	(102 + 34) mL
				33.5 mL	
910FM	900 g	675 g	225 g	Stachement 910 FM (Stachema, CZ)	(107 + 35) mL
				27.2 mL	
WR2	900 g	675 g	225 g	WR-2 (Sika, CHE)	(116 + 39) mL
				14.4 mL	
PC2	900 g	675 g	225 g	PC-2 (Sika, CHE)	(116 + 39) mL
				14.4 mL	
20HE	900 g	675 g	225 g	20HE (Sika, CHE)	(113 + 38) mL
				18.0 mL	

**Table 2:** Composition of all mixtures for optimization regarding the contents of silica fume and superplasticizer.

Mixture Designation	Fine Sand According to ČSN EN 196-1 [39] (Filtrační písky Chlum, CZ)	CEM I 52.5 R-SR 5 White (Aalborg Portland, DE)	Silica Fume RW Füller-Q (Elkem, D)	Superplasticizer MasterGlenium ACE 446 (BASF, D)	Demineralized Water
SF10/SP2.0	900 g	810 g	90 g	18 mL	(125 + 41) mL
SF15/SP2.0	900 g	765 g	135 g	18 mL	(125 + 41) mL
SF20/SP2.0	900 g	720 g	180 g	18 mL	(125 + 41) mL
SF25/SP2.0	900 g	675 g	225 g	18 mL	(125 + 41) mL
SF30/SP2.0	900 g	630 g	270 g	18 mL	(125 + 41) mL
SF35/SP2.0	900 g	585 g	315 g	18 mL	(125 + 41) mL
SF10/SP2.5	900 g	810 g	90 g	22.5 mL	(122 + 40) mL
SF15/SP2.5	900 g	765 g	135 g	22.5 mL	(122 + 40) mL
SF20/SP2.5	900 g	720 g	180 g	22.5 mL	(122 + 40) mL
SF25/SP2.5	900 g	675 g	225 g	22.5 mL	(122 + 40) mL
SF30/SP2.5	900 g	630 g	270 g	22.5 mL	(122 + 40) mL
SF35/SP2.5	900 g	585 g	315 g	22.5 mL	(122 + 40) mL
SF10/SP3.0	900 g	810 g	90 g	27 mL	(119 + 39) mL
SF15/SP3.0	900 g	765 g	135 g	27 mL	(119 + 39) mL
SF20/SP3.0	900 g	720 g	180 g	27 mL	(119 + 39) mL
SF25/SP3.0	900 g	675 g	225 g	27 mL	(119 + 39) mL
SF30/SP3.0	900 g	630 g	270 g	27 mL	(119 + 39) mL
SF35/SP3.0	900 g	585 g	315 g	27 mL	(119 + 39) mL
SF10/SP3.5	900 g	810 g	90 g	31.5 mL	(117 + 38) mL
SF15/SP3.5	900 g	765 g	135 g	31.5 mL	(117 + 38) mL
SF20/SP3.5	900 g	720 g	180 g	31.5 mL	(117 + 38) mL
SF25/SP3.5	900 g	675 g	225 g	31.5 mL	(117 + 38) mL
SF30/SP3.5	900 g	630 g	270 g	31.5 mL	(117 + 38) mL
SF35/SP3.5	900 g	585 g	315 g	31.5 mL	(117 + 38) mL
SF10/SP4.0	900 g	810 g	90 g	36 mL	(114 + 37) mL
SF15/SP4.0	900 g	765 g	135 g	36 mL	(114 + 37) mL
SF20/SP4.0	900 g	720 g	180 g	36 mL	(114 + 37) mL
SF25/SP4.0	900 g	675 g	225 g	36 mL	(114 + 37) mL
SF30/SP4.0	900 g	630 g	270 g	36 mL	(114 + 37) mL
SF35/SP4.0	900 g	585 g	315 g	36 mL	(114 + 37) mL
SF10/SP4.5	900 g	810 g	90 g	40.5 mL	(110 + 37) mL
SF15/SP4.5	900 g	765 g	135 g	40.5 mL	(110 + 37) mL
SF20/SP4.5	900 g	720 g	180 g	40.5 mL	110 + 37) mL
SF25/SP4.5	900 g	675 g	225 g	40.5 mL	(110 + 37) mL
SF30/SP4.5	900 g	630 g	270 g	40.5 mL	(110 + 37) mL
SF35/SP4.5	900 g	585 g	315 g	40.5 mL	(110 + 37) mL

## Data Availability

The data presented in this study are available upon request from the corresponding authors.
